# Nocturnal blood pressure is associated with sympathetic nerve activity in patients with chronic kidney disease

**DOI:** 10.14814/phy2.14602

**Published:** 2020-10-28

**Authors:** Jin H. Jeong, Ida T. Fonkoue, Arshed A. Quyyumi, Dana DaCosta, Jeanie Park

**Affiliations:** ^1^ Division of Renal Medicine Department of Medicine Emory University School of Medicine Atlanta GA USA; ^2^ Division of Cardiology Department of Medicine Emory University School of medicine Atlanta GA USA; ^3^ Department of Veterans Affairs Health Care System Research Service Line Decatur GA USA

**Keywords:** Chronic Kidney Disease, Endothelial Dysfunction, Nocturnal Blood Pressure, Sympathetic Nervous System

## Abstract

Elevated nocturnal blood pressure (BP) and nocturnal non‐dipping are frequently observed in patients with chronic kidney disease (CKD) and are stronger predictors of cardiovascular complications and CKD progression than standard office BP. The sympathetic nervous system (SNS) is thought to modulate diurnal hemodynamic changes and the vascular endothelium plays a fundamental role in BP regulation. We hypothesized that SNS overactivity and endothelial dysfunction in CKD are linked to elevated nocturnal BP and non‐dipping. In 32 CKD patients with hypertension (56 ± 7 years), office BP, 24‐hr ambulatory BP, muscle sympathetic nerve activity (MSNA) and endothelial function via flow‐mediated dilation (FMD) were measured. Participants were subsequently divided into dippers (nighttime average BP > 10% lower than the daytime average BP, *n* = 8) and non‐dippers (*n* = 24). Non‐dippers had higher nighttime BP (*p* < .05), but not office and daytime BP, compared to dippers. MSNA burst incidence (81 ± 13 versus 67 ± 13 bursts/100 HR, *p* = .019) was higher and brachial artery FMD (1.7 ± 1.5 versus 4.7 ± 1.9%, *p* < .001) was lower in non‐dippers compared to dippers. MSNA and FMD each predicted nighttime systolic (β = 0.48,‐0.46, *p* = .02, 0.07, respectively) and diastolic BP (β = 0.38,‐0.47, *p* = .04, 0.03, respectively) in multivariate‐adjusted analyses. Our novel findings demonstrate that unfavorable nocturnal BP profiles are associated with elevated SNS activity and endothelial dysfunction in CKD. Specifically, CKD patients with higher nighttime BP and the non‐dipping pattern have higher MSNA and lower FMD. These support our hypothesis that SNS overactivation and endothelial dysfunction are linked to the dysregulation of nighttime BP as well as the magnitude of BP lowering at nighttime in CKD.

## Graphical abstract

Elevated nocturnal blood pressure (BP) and nocturnal non‐dipping are frequently observed in patients with chronic kidney disease (CKD) and are stronger predictors of cardiovascular complications and CKD progression than standard office BP. Our novel findings demonstrate that unfavorable nocturnal BP profiles are associated with elevated SNS activity and endothelial dysfunction, supporting our hypothesis that SNS overactivation and endothelial dysfunction are linked to dysregulation of nighttime BP as well as the magnitude of BP lowering at nighttime in CKD.

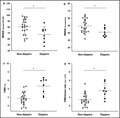

## 1 INTRODUCTION

Chronic kidney disease (CKD) is characterized by a high prevalence of comorbid hypertension (HTN) and abnormal blood pressure (BP) patterns that are both associated with increased risk of cardiovascular disease (CVD) and mortality (Muntner et al., 2010). Ambulatory blood pressure monitoring (ABPM) allows for the collection of diurnal variations of BP over a 24‐hr period and is superior to clinic BP measurements for the prediction of cardiovascular outcomes Verdecchia et al., (1994). A non‐dipping pattern characterized by the loss of the normal BP drop of 10%–20% during nighttime from daytime, as well as reverse dipping or nocturnal hypertension, are frequently observed and associated with worse prognosis in CKD (Iimuro et al., (2013); Pogue et al., 2009). Furthermore, non‐dipping is shown to be an independent risk factor for subclinical target organ damage including coronary artery calcification (Choi et al., 2017) and cardiac hypertrophy (Jaques et al., 2018), and is a significant predictor of CVD and mortality in patients with CKD (Wang et al., 2016). Despite the clinical significance of non‐dipping BP, the underlying mechanisms responsible for aberrant ABPM patterns in CKD are poorly understood.

One potential mechanism contributing to non‐dipping BP in CKD is the chronic overactivation of the sympathetic nervous system (SNS) (Park, 2012). Prior evidence demonstrates that the autonomic nervous system is involved in the regulation of circadian cardiovascular rhythms and increased SNS activation may lead to impaired diurnal BP variation. In support of this contention, the normal diurnal dipping pattern was lost in patients with autonomic failure (Carvalho et al., 2000) and with quadriplegia from cervical spinal cord injuries (Munakata et al., 1997). In normotensive individuals, non‐dippers were shown to have reduced nighttime fall in both norepinephrine and epinephrine excretion rates as well as heighted α‐adrenergic receptor responsiveness compared to dippers (Sherwood et al., 2002). Similarly, hypertensive non‐dippers were reported to have elevated plasma catecholamine levels (Kohara et al., 1995) and altered sympathovagal balance (Hojo et al., 1997), suggesting a mechanistic link between SNS overactivity and non‐dipping BP patterns. Although CKD patients have chronic SNS overactivation (Park, 2012) and heightened vascular α‐adrenergic receptor sensitivity (Sprick et al., 2019), the role of SNS overactivation in modulating nighttime BP elevation in CKD has not been previously investigated.

CKD is also associated with reduced endothelial nitric oxide (NO) synthesis (Martens et al., 2016) that could contribute to increased nighttime BP both directly via endothelial dysfunction and indirectly via increased SNS activation. Prior studies have shown endothelial vascular function is more impaired in hypertensive patients with a non‐dipping BP pattern compared to dippers (Higashi et al., 2002). In postmenopausal women, endothelial dysfunction was associated with elevated nighttime BP (Routledge et al., 2015). In addition to its important role in the local regulation of peripheral vascular tone, NO has a tonic inhibitory effect on central SNS activity (Charkoudian et al., 2006). Therefore, a reduction in NO bioavailability could lead not only to attenuated endothelium‐dependent vasodilation but also reduced the inhibition of sympathetic outflow resulting in increased BP. These previous findings suggest that decreased NO bioavailability, frequently reported in CKD (Downey et al., 2017; Kuczmarski et al., 2011), might be involved in the blunted nighttime BP drop. However, the link between vascular endothelial function, SNS activation, and nocturnal BP regulation in CKD is unknown.

Therefore, we aimed to investigate the potential mechanisms underlying abnormal nocturnal BP regulation in patients with CKD. We hypothesized that SNS overactivity and endothelial dysfunction are linked to elevated nocturnal BP and non‐dipping pattern in patients with CKD.

## 2 METHODS

### 2.1 Study population

Thirty‐two participants with CKD and age‐matched hypertensive controls without CKD were recruited and enrolled from outpatient clinics at the Atlanta Veterans Affairs (VA) Healthcare System. All CKD participants had a confirmed diagnosis of either Stage 2 [estimated glomerular filtration rate (eGFR) of 60–89 ml·min^−1^.1.73 m^−2^ with concomitant urinary microalbumin: creatinine ratio of > 30 mg/g] or Stage 3 (eGFR of 30–59 ml·min^−1^.1.73 m^−2^) CKD (Levey et al., 2006). CKD participants had at least a 3‐month history of stable kidney function (≤10% fluctuation in eGFR) and stable antihypertensive medication regimen before enrollment. Exclusion criteria included severe CKD (eGFR < 30 cm·l^−1^·min^−1^); diabetes; HIV infection; clinical evidence of chronic heart failure or ejection fraction below 35%; any history of past coronary artery, cerebrovascular, aortic, or peripheral vascular disease; symptomatic heart disease determined by electrocardiogram, stress test, and/or history; hepatic enzyme concentrations greater than two times the upper limit of normal; severe anemia with hemoglobin level < 10 g/dl; history of nephrolithiasis; any serious systemic disease that might influence survival; current treatment with clonidine, levodopa, or methotrexate; clinic BP greater than 160/90 mmHg; office BP less than 110/60; change in medications or surgery within the past 3 months; drug or alcohol abuse.

### 2.2 Study design

After written informed consent was obtained, office BP, 24‐hr ABPM, basic metabolic panel, and urinary microalbumin and creatinine levels were obtained during a screening visit. During two separate study visits, brachial artery flow mediated‐dilation (FMD) and muscle sympathetic nervous activity (MSNA) by microneurography were obtained. All measurements were obtained in a quiet, temperate (21°C) environment between 8 and 10 a.m., after abstaining from food, caffeine, smoking, and alcohol for at least 12 hr, and exercise for at least 24 hr. All participants reported having taken prescribed medications as normally directed. This study was approved by the Emory University Institutional Review Board and the Atlanta VA Healthcare System Research and Development Committee.

### 2.3 Measurements and procedures

#### 2.3.1 Blood Pressure

Office BP was measured after 5 min of rest in a seated position with the arm supported at heart level using an appropriately sized cuff per American College of Cardiology/American Heart Association (ACC/AHA) guidelines (Whelton et al., 2018). All BP measurements were taken by a single‐study coordinator with an automated device (Omron, HEM‐907XL, Omron Healthcare, Kyoto, Japan) using the oscillometric method. Each data point of BP was obtained as an average of three consecutive BP measurements separated by 5 min. Mean arterial blood pressures (MAP) was calculated as 2/3 diastolic BP (DBP) + 1/3 systolic BP (SBP).

#### 2.3.2 24‐Hour Ambulatory Blood Pressure Monitoring

Participants were instrumented with the validated noninvasive oscillometric Spacelabs 90,227 ABPM device (Spacelabs Healthcare, WA, USA) which is the most commonly used device for ABPM monitoring meeting the validation criteria of the British Hypertension Society ( O'Brien et al., 2003). Cuff size was chosen based on arm circumference and fixed to the nondominant arm by a single‐study investigator.

SBP and DBP were measured every 20 min during the daytime and every 30 min during the nighttime over a consecutive 24‐hr period. Wake and sleep periods, defined by self‐report, were used to compute mean daytime BP and mean nighttime BP values, respectively. Artifactual ABPM readings were identified by the Spacelabs software. Participants were asked to attend to their usual activities and avoid strenuous physical activity. Participants were instructed to keep motionless at the time of each measurement. Participants had no access to ABPM values. The average BP reading success rate was 91.2 ± 7.6%. ABPM data had recordings of good technical quality (with at least 70% valid readings during the 24‐hr period, at least 20 valid readings, while awake with at least two valid readings per hour and at least seven valid readings, while asleep with at least one valid reading per hour), in line with the minimum requirements recommended by the European Society of Hypertension (O'Brien et al., 2013). The participants were further divided into dippers (nighttime MAP average > 10% lower than the daytime MAP average, *n* = 8) and non‐dippers (*n* = 24) for group comparison analyses.

#### 2.3.3 Muscle sympathetic nerve activity

Multiunit postganglionic MSNA was recorded directly from the peroneal nerve by microneurography, as previously described (Wallin & Fagius, 1988). Participants were placed in a supine position, and the leg was positioned for microneurography. A tungsten microelectrode (tip diameter 5–15 μm) (Bioengineering, University of Iowa) was inserted into the nerve, and a reference microelectrode was inserted subcutaneously 1–2 cm from the recording electrode. The signals were amplified (total gain: 50,000–100,000), filtered (700–2,000 Hz), rectified, and integrated (time constant 0.1 s) to obtain a mean voltage display of sympathetic nerve activity (Nerve Traffic Analyzer, model 662C‐4, University of Iowa, Bioengineering) that was recorded by the LabChart 7 Program (PowerLab 16sp, ADInstruments). Continuous ECG was recorded simultaneously with the neurogram using a BioAmp system. Beat‐to‐beat arterial BP was measured concomitantly using a noninvasive monitoring device that detects digital blood flow via finger cuffs and translates blood flow oscillations into continuous pulse pressure waveforms and beat‐to‐beat values of BP (CNAP, CNSystems) (Jeleazcov et al., 2010). Absolute values of BP were internally calibrated using a concomitant upper arm BP reading and were calibrated at the start and every 15 min throughout the study. The tungsten microelectrode was manipulated to obtain a satisfactory nerve recording that met previously established criteria (Mano et al., 2006). After 10 min of rest, baseline BP, heart rate (HR), respiratory rate, and MSNA were recorded continuously for 10 min.

#### 2.3.4 Brachial artery flow‐mediated dilation

A forearm occlusion cuff was placed on participants while they were in a supine position. A 13‐MHz high‐resolution ultrasound transducer (Acuson Aspen) was used longitudinally 2–10 cm above the antecubital fossa to record brachial artery measurements. Baseline values were obtained by averaging diameter and blood velocity over three cardiac cycles measured via ECG gating to capture end‐diastolic arterial diameters. The forearm cuff was inflated to suprasystolic levels (50 mmHg above SBP) using a rapid cuff inflator (D.E. Hokanson) for 5 min. Peak hyperemic blood velocity was measured by Doppler ultrasound during the first 10 s following cuff release. Diameter measurements were obtained 60 and 90 s following cuff release. FMD calculations were made using 60‐s and 90‐s measurements to determine peak diameter. Arterial diameters were measured and analyzed by a single investigator blinded to the clinical status of the participant from digitized images utilizing customized software (Medical Imaging Applications). FMD is given as the percent change in artery diameter from baseline: (peak hyperemic diameter – baseline diameter)/baseline diameter. Shear rate at baseline and peak hyperemia was calculated as 4 × peak blood velocity/arterial diameter. FMD values were then normalized for peak hyperemic shear rate. This calculation of the shear rate is consistent with our previous reports (Park et al., 2015).

### 2.4 Data Analysis

#### 2.4.1 Sample size calculation

A power analysis was conducted using estimates from a relevant study (Grassi et al., 2008) in a different patient population of hypertensives that found a significant group difference in MSNA burst incidence between dippers and non‐dippers (55.6 ± 5.3 and 67.5 ± 10.3 bursts/100 HR, *p* < .05) as well as a significant linear association between MSNA and the difference of day to night SBP (r= −0.76, *p* < .001. In addition, Higashi et al. (2002) demonstrated a smaller response of forearm blood flow to intra‐arterial acetylcholine, an endothelium‐dependent vasodilator, in non‐dippers compared to dipper hypertensive patients (25.1 ± 13.8 versus. 20.2 ± 13.4 ml min^‐1^ 100 ml^‐1^ of tissue, *p* < .05). Based on these estimates, enrolling 32 participants had > 80% power with an α of 0.05 to detect group differences and linear associations if they truly exist.

#### 2.4.2 Muscle sympathetic nerve activity

MSNA and ECG data were exported from the LabChart data acquisition system to WinCPRS (Absolute Aliens, Turku, Finland) for analysis. R‐waves were detected and marked from the continuous ECG recording. MSNA bursts were automatically detected by the program using the following criteria: 3:1 burst‐to‐noise ratio within a 0.5‐s search window, with an average latency in burst occurrence of 1.2–1.4 s from the previous R‐wave. After automatic detection, the ECG and MSNA neurograms were visually inspected for accuracy of detection by a single investigator (J. Park). MSNA was expressed as burst incidence (bursts/100 heartbeats) and burst frequency (bursts/min).

#### 2.4.3 Statistics

Values are presented as mean ± standard deviation (*SD*) unless otherwise noted. Differences in participant characteristics between dippers and non‐dippers were determined using independent *t*‐tests for continuous variables or chi‐square tests for categorical variables. The group difference in primary outcomes (MSNA, FMD) was tested by independent *t*‐tests. In a secondary analysis, one way‐ANOVA analysis was performed to examine the 3‐group comparison in primary outcomes with controls, CKD‐dippers and CKD‐nondippers followed by LSD‐post hoc tests. Pearson correlation test was performed to examine the linear relationship between BP variables and MSNA and FMD. Multivariable linear regression tests were used to describe the predictability of MSNA and FMD on nighttime BP variables after controlling for age and body mass index (BMI) and smoking status as covariates (Table 4 *, Model 1 ~ 6)*. To determine the relative association of office, daytime, and nighttime BP with MSNA and FMD, linear regression models predicting MSNA or FMD were run with the different BP initially entered as predictors (Table 4 *, Model 7 ~ 12*). The only predictor variable(s) retained in the regression models after the step‐wise variable selection method (*p* = .1 for removal) indicates a higher degree of relevance with the dependent variable.

The indirect effect was tested by mediation analysis using a percentile bootstrap estimation approach with 5,000 samples, implemented with the PROCESS macro Version 3 (Hayes & Rockwood, 2017). This examines whether MSNA (or FMD) mediates the effect of FMD (or MSNA) on nighttime BP with adjustment for age and BMI. An α < 0.05 was considered statistically significant for all analyses. All analyses were performed using SPSS version 26.0 (IBM Corporation, Somers, NY).

## 3 RESULTS

### 3.1 Participant Characteristics

Thirty‐two participants with CKD who met the eligibility criteria were enrolled in the study. Table 1 shows the characteristics of the study participants. The majority of participants were male and African American. The main causes of CKD were HTN (*n* = 14), an unknown cause combined with HTN (*n* = 5), polycystic kidney disease (*n* = 2), glomerulonephritis (*n* = 1) and unknown (*n* = 10). All participants in both groups had HTN. There were no significant differences in demographics, anthropometric characteristics, proportion of smokers, obstructive sleep apnea (OSA) or antihypertensive medication use, or HMG CoA reductase inhibitors (statins) between dippers and non‐dippers with CKD.

**Table 1:** Participant Characteristics

Characteristics	Dippers (*n* = 8)	Non‐dippers (*n* = 24)	*p*‐value
Age (yr)	58.0 ± 6.4	55.0 ± 7.6	.318
Gender (male, %)	8 (100%)	24 (100%)	.226
Race			.056
African Americans	6 (75%)	24 (100%)	
Caucasian	2 (25%)	0	
Height (cm)	181 ± 10	179 ± 6	.538
Weight (kg)	106 ± 27	106 ± 15	.952
BMI (kg/m^2^)	32.1 ± 6.3	33.0 ± 4.4	.537
Hypertension (*n*)	24	8	1.000
Smokers (*n*)	0	7	.146
OSA (*n*)	4	2	.625
Antihypertensive Medications (*n*,%)			
Calcium channel blockers	2 (25%)	16 (67%)	.096
ACEI/ARB	7 (88%)	16 (67%)	.386
Diuretics	3 (38%)	12 (50%)	.691
β‐blockers	1 (13%)	9 (38%)	.380
Aldosterone receptor blockers	1 (1%)	2 (8%)	1.000
α‐blockers	1 (1%)	2 (8%)	1.000
Hydralazine	0	2 (8%)	1.000
Statins (*n*,%)	2 (25%)	6 (25%)	1.000
eGFR (ml·min^−1^.1.73 m^−2^)	48 ± 13	52 ± 14	.412
Blood Glucose (mg/dL)	104 ± 27	93 ± 13	.139
UACR (μg/mg)	186 ± 260	142 ± 209	.633

Note

Values are expressed as means ± standard deviation.

Abbreviations: BMI, Body Mass Index; OSA, obstructive Sleep Apnea; eGFR, Estimated Glomerular Filtration Rate; UACR, Urinary Albumin to Creatinine Ratio.

### 3.2 Ambulatory Blood Pressure between Dippers and Non‐dippers in Patients with CKD

There was no difference in office and daytime BP variables between dippers and non‐dippers in patients with CKD (Table 2). However, nighttime mean and minimum BP values were higher in the non‐dippers compared to the dippers. There were no differences in nighttime HR between groups. There was less % dipping of SBP, DBP, MAP, but not HR in the non‐dippers compared to dippers.

**Table 2:** The Office, averaged Daytime, and Nighttime Hemodynamic Parameters between Dippers and Non‐dippers in Patients with CKD

Characteristics	Dippers (*n* = 8)	Non‐dippers (*n* = 24)	*p*‐value
Office Hemodynamics			
Rest SBP (mmHg)	133.6 ± 9.4	138.7 ± 12.6	.327
Rest DBP (mmHg)	84.6 ± 9.8	86.4 ± 7.7	.603
Rest MAP (mmHg)	100.9 ± 9.1	103.9 ± 8.2	.420
Rest HR (bpm)	63.7 ± 8.7	64.5 ± 8.2	.821
Daytime Hemodynamics			
Daytime SBP (mmHg)	132.0 ± 13.0	132.2 ± 10.6	.971
Daytime DBP (mmHg)	81.4 ± 8.6	80.8 ± 7.5	.865
Daytime MAP (mmHg)	97.8 ± 9.5	97.6 ± 8.2	.972
Daytime HR (bpm)	72.5 ± 6.8	73.2 ± 12.3	.879
Nighttime Hemodynamics			
Nighttime SBP (mmHg)	115.5 ± 13.6	131.5 ± 13.0	**.006**
Nighttime DBP (mmHg)	69.1 ± 9.9	79.9 ± 8.7	**.007**
Nighttime MAP (mmHg)	83.8 ± 10.3	97.0 ± 9.7	**.003**
Nighttime HR (bpm)	64.8 ± 10.9	69.0 ± 8.5	.265
Nighttime minSBP (mmHg)	99.5 ± 12.6	114.5 ± 13.8	**.011**
Nighttime minDBP (mmHg)	55.3 ± 8.8	64.3 ± 10.7	**.040**
Nighttime minMAP (mmHg)	70.8 ± 7.6	81.8 ± 10.7	**.012**
Nighttime minHR (bpm)	56.3 ± 8.6	61.1 ± 6.7	.111
Dipping Parameters			
SBP dipping (%)	12.5 ± 5.6	0.6 ± 4.6	**<.001**
DBP dipping (%)	15.3 ± 4.2	1.3 ± 4.9	**<.001**
MAP dipping (%)	14.5 ± 3.8	0.7 ± 4.6	**<.001**
HR dipping (%)	11.0 ± 10.2	4.9 ± 8.2	.201

Note

Values are expressed as mean ± standard deviation. Significant p‐values are shown in bold.

Abbreviations: CKD, Chronic Kidney Disease; DBP: Brachial Diastolic Blood Pressure; HR: Heart Rate; MAP: Mean Arterial Blood Pressure; minDBP: minimum DBP; minMAP, minimum MAP; minSBP, minimum SBP; SBP, Systolic Blood Pressure.

### 3.3 MSNA and FMD between dippers and Non‐dippers in patients with CKD and controls

**Figure 1:**
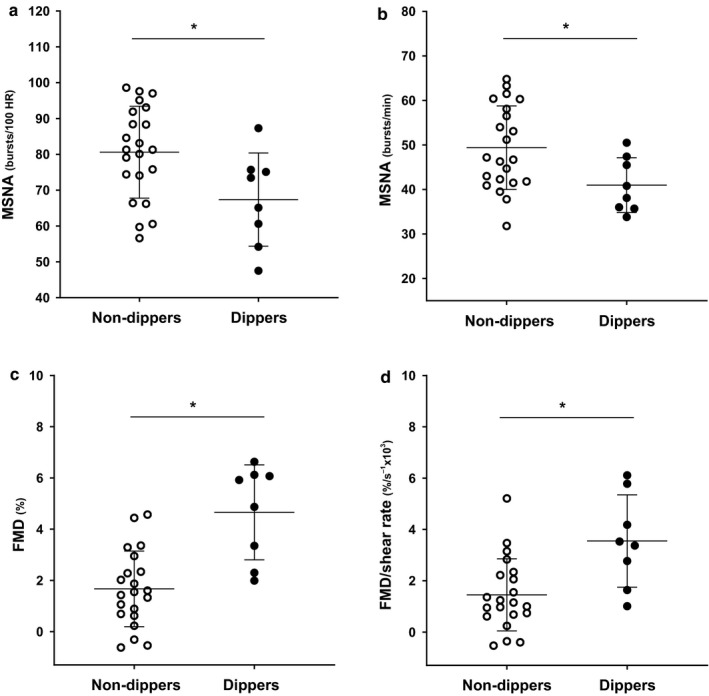
Individual data and group means (± standard deviation) in muscle sympathetic nerve activity (MSNA) burst incidence (a) and burst frequency (b), and brachial artery flow‐mediated dilation (FMD) (c), and FMD corrected for shear rate (d) between Dippers (*N* = 8) and Non‐dippers (*N* = 24) in patients with chronic kidney disease.* indicates a significant difference (*p* < .05) between groups by *t* tests

MSNA burst incidence (81 ± 13 versus. 67 ± 13 bursts/100 HR, *p* = .019) and burst frequency (49 ± 9 versus. 40 ± 6 bursts/min, *p* = .026) were higher in non‐dippers compared to dippers with CKD (*Figure* *1a* *‐* *b*). Likewise, brachial artery FMD (1.7 ± 1.5 versus. 4.7 ± 1.9%, *p* < .001) and FMD corrected for shear stress (1.5 ± 1.4 versus. 3.5 ± 1.8%/s^‐1^x10^3^, *p* = .003) was significantly lower in non‐dippers compared to dippers (*Figure* *1c* *‐* *d*, Table 3).

**Table 3:** FMD Parameters between Dippers and Non‐dippers in Patients with CKD

	Dippers (*n* = 8)	Nondippers (*n* = 24)	*p*‐value
FMD (%)	4.7 ± 1.9	1.7 ± 1.5	**<.001**
FMD/shear (%/s^−1^x10^3^)	3.5 ± 1.8	1.5 ± 1.4	**.003**
Diameter‐baseline (mm)	4.2 ± 0.5	4.5 ± 0.5	.113
Diameter‐hyperemic (mm)	4.4 ± 0.5	4.6 ± 0.5	.287
Blood velocity‐baseline (m/s)	0.91 ± 0.39	0.85 ± 0.32	.707
Blood velocity‐hyperemic (m/s)	1.47 ± 0.26	1.34 ± 0.25	.255
Shear rate‐baseline (s^−1^x10^3^)	0.93 ± 0.53	0.77 ± 0.33	.364
Shear rate‐hyperemic (s^−1^x10^3^)	1.38 ± 0.32	1.18 ± 0.26	.098

Note.

Significant p‐values are shown in bold.

Abbreviation: FMD, Flow‐Mediated dilation.

In a secondary analysis that included data from six sex‐, age‐, and race‐matched hypertensive controls without kidney disease (51 ± 7 yr, BMI: 30.7 ± 3.9 kg/m^2^) for a 3‐group comparison, there was a significant group effect in MSNA burst incidence (*F*(2,33)=10.2, *p* < .001), burst frequency (*F*(2,33)=8.8, *p* = .001), FMD (*F*(2,32)=12.1, *p* < .001) and FMD/shear (*F*(2,32)=6.5, *p* = .004). Post hoc tests showed that MSNA burst incidence (51.2 ± 22.0 bursts/100 HR) and burst frequency (30 ± 16 bursts/min) in controls were lower compared to CKD‐dippers (*p* = .049, *p* = .06, respectively) and CKD‐nondippers (*p* < .001 for both), while FMD (7.8 ± 6.6%), FMD/shear (3.4 ± 2.2%/s^‐1^x10^3^) in controls was similar to CKD‐dippers (*p* > .05 for both) but higher than CKD non‐dippers (*p* < .001, *p* = .014, respectively).

### 3.4 Linear Association of MSNA and FMD and ABPM in Patients with CKD

#### 3.4.1 MSNA and BP variables

**Figure 2:**
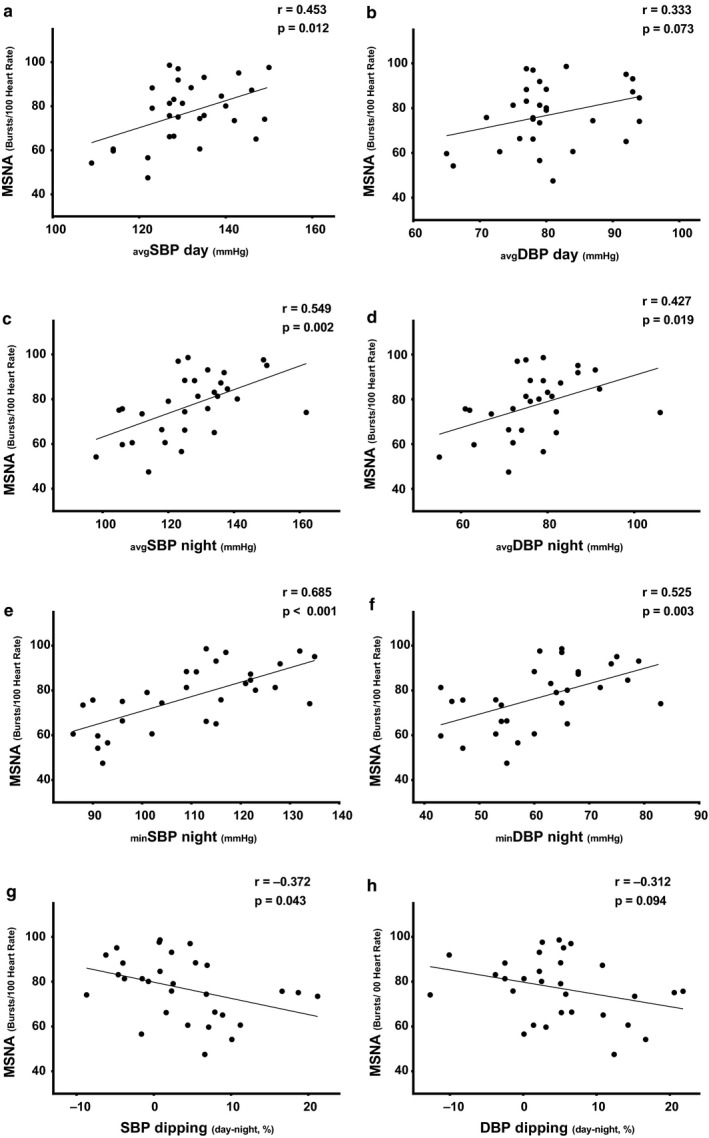
The association between muscle sympathetic nerve activity (MSNA) and (a) average (avg) daytime systolic blood pressure (SBP), (b) avg daytime diastolic BP (DBP), (c) avg nighttime SBP, (d) avg nighttime DBP, (e) minimum (min) nighttime SBP, (f) min nighttime DBP, (g) SBP dipping, and (h) DBP dipping. *N* = 32 for all analyses

Daytime, nighttime, and nighttime‐minimum BP variables and the magnitude of BP dipping were significantly associated with MSNA burst incidence and burst frequency in patients with CKD (*p* < .05 for all except daytime DBP, *Figure* *2* *a~h*). In contrast, there was no significant association between office BP and MSNA burst incidence or burst frequency (*p* > .05 for both).

Multivariable linear regression analysis demonstrated that MSNA burst incidence significantly predicts nighttime SBP, DBP, and MAP after adjusting for age, BMI, and smoking status (*Model 1–3,* Table 4) or after adjusting for OSA (*p* < .05 for all). No significant association was found between any of the outcome variables (MSNA and nighttime BP) and any of the covariates (age, BMI, smoking status, and OSA).

**Table 4:** Multivariable Regression Analysis with Office, Daytime, and Nighttime BP Variables for MSNA and FMD in Patients with CKD

Model	*Multivariable Linear Regression Analysis*								
	Dependent Variable	Predictor	Covariates		Unstandardized β	Standardized β	R^2^	Adj R^2^	Model P‐value
1	Night SBP	MSNA	Age, BMI, Smoking		0.508	0.484	0.376	0.272	**0.019**
2	Night DBP	MSNA	Age, BMI, Smoking		0.279	0.380	0.334	0.223	**0.038**
3	Night MAP	MSNA	Age, BMI, Smoking	0.360		0.445	0.352	0.244	**0.029**
4	Night SBP	FMD	Age, BMI, Smoking	−2.54		−0.464	0.495	0.176	0.070
5	Night DBP	FMD	Age, BMI, Smoking	−1.820		−0.470	0.361	0.254	**0.025**
6	Night MAP	FMD	Age, BMI, Smoking	−2.170		−0.507	0.354	0.246	**0.028**

	*Stepwise Multivariable Linear Regression Analysis*							
	Dependent Variable	Predictors				R^2^	Adj R^2^	Model P‐value
		Entered	Selected	Unstandardized β	Standardized β			
7	MSNA	Office SBP Day SBP Night SBP	Night SBP	0.449	0.450	0.203	0.172	**0.016**
8	MSNA	Office DBP Day DBP Night DBP	None					
9	MSNA	Office MAP Day MAP Night MAP	Night MAP	0.550	0.381	0.145	0.112	**0.045**
10	FMD	Office SBP Day SBP Night SBP	Night SBP	−0.086	−0.564	0.318	0.291	**0.002**
11	FMD	Office DBP Day DBP Night DBP	Night DBP	−0.131	−0.613	0.376	0.351	**0.001**
12	FMD	Office MAP Day MAP Night MAP	Night MAP	−0.121	−0.616	0.379	0.355	**0.001**

Note

Significant *p*‐values are shown in bold.

Abbreviations: BP, Blood Pressure; CKD, Chronic Kidney Disease; DBP, Diastolic Blood Pressure; FMD, Flow‐Mediated dilation; MAP, Mean Arterial Blood Pressure; MSNA, Muscle Sympathetic Nervous Activity; SBP, Systolic Blood Pressure.

We then considered different BP variables (daytime, nighttime, and office BP) as potential predictors of MSNA burst incidence. As opposed to daytime SBP and office SBP, only nighttime SBP remained as a significant predictor of MSNA burst incidence in the multivariable linear regression model with a stepwise variable selection method (*Model 7,* Table 4). Similarly, only nighttime MAP (and not daytime MAP or office MAP) remained as a significant predictor of MSNA burst incidence in the same model (*Model 9,* Table 4).

#### 3.4.2 FMD and BP variables

**Figure 3:**
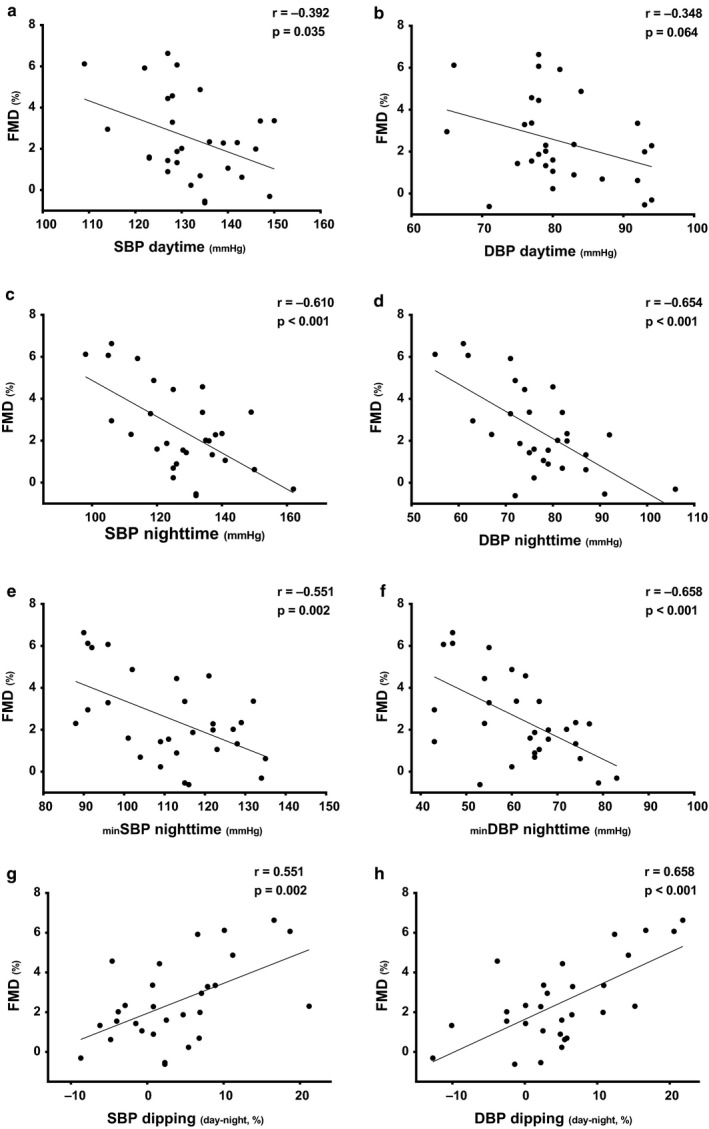
The association between flow‐mediated dilation (FMD) and daytime and nighttime hemodynamics. (a) Average (avg) daytime systolic blood pressure (SBP), (b) avg daytime diastolic BP (DBP), (c) avg nighttime SBP, (d) avg nighttime DBP, (e) minimum (min) nighttime SBP, (f) min nighttime DBP, (g) SBP dipping, and (h) DBP dipping. *N* = 32 for all analyses

Daytime, nighttime, and nighttime‐minimum BP variables, the magnitude of dipping and office BP were associated with FMD (*p* < .05 for all) (*Figure* *3* *a~h*).

Multivariable linear regression analysis demonstrated that the variability of nighttime BP is in part explained by FMD after adjusting for age, BMI, and smoking status (*Model 4 ~ 6,* Table 4) or after adjusting for OSA (*p* < .05 for all).

When considering daytime, nighttime, and office SBP and MAP as potential predictors, only nighttime BP remained as the only significant predictor (over daytime and office BP) for FMD (*Model 10 ~ 12,* Table 4).

### 3.5 Mediating effect of MSNA and FMD on nighttime BP

MSNA burst incidence and burst frequency were significantly associated with FMD (*r* = −0.572, −0.578, respectively, *p* < .001 for both) and FMD/shear rate (*r* = −0.510, −0.546, respectively, *p* = .003, 0.006, respectively). Regression‐based mediation analysis showed that approximately 31% of the variation in nighttime SBP was accounted by the combination of two predictors, FMD and MSNA burst incidence (*r* ^2^ = 0.312, *p* = .016) or FMD and MSNA burst frequency (*r* ^2^ = 0.203, *p* = .05), after adjustment for age and BMI. FMD was no longer a significant predictor for nighttime SBP (β=−1.28, *p* = .26) after controlling for MSNA burst incidence as a mediator and age and BMI as covariates, while FMD remained marginally significant after controlling for MSNA burst frequency, age, and BMI as mediators (β=−2.10, *p* = .099). The indirect effect of MSNA burst incidence and burst frequency explained 46.9% and 13.3% of the relationship between FMD and nighttime SBP (indirect effect size: −1.13 and 0.32, respectively; 95%CI= (−3.00, 0.49) and (−1.70, 1.08), respectively) after adjusting for age and BMI (Table 4).

Moreover, when FMD was considered as a mediator, MSNA burst incidence was still a marginally significant predictor for nighttime SBP (β = 0.366, *p* = .097), but not with MSNA burst frequency (β = 0.140, *p* = .676). The indirect effect of FMD explained 28.2% and 71.7% of the relationship between nighttime SBP and MSNA burse incidence and burst frequency, respectively (indirect effect size: 0.14 and 0.36, respectively; 95%CI= (−0.13, 0.48) and (−0.01, 0.82), respectively) after adjusting for age and BMI.

## 4 DISCUSSION

Nocturnal HTN and non‐dipping pattern of BP are common in patients with CKD (Iimuro et al., 2013; Pogue et al., 2009) and are linked to an increased risk of CVD and mortality (Choi et al., 2017; Jaques et al., 2018; Wang et al., 2016). Identifying the underlying mechanisms may enable the development of strategies to mitigate elevated nighttime BP and potentially attenuate future CVD risk. The present study demonstrates that unfavorable nocturnal BP profiles are associated with elevated SNS activation and impaired vascular endothelial function in patients with CKD. Specifically, we report for the first time that CKD patients with higher nighttime BP and non‐dipping BP patterns have higher levels of MSNA and lower FMD compared to CKD patients with lower nighttime BP and intact nocturnal dipping, despite similar measures of both daytime BP and office BP between the groups. These findings support our hypothesis that basal sympathetic overactivation and vascular endothelial dysfunction may be involved in the dysregulation of nighttime BP as well as the blunted magnitude of BP lowering at night in CKD patients.

HTN is highly prevalent in CKD and is the leading risk factor for mortality in this patient population (Muntner et al., 2010). ABPM provides detailed information about BP diurnal variation and has been shown to be a better predictor of target‐organ damage and cardiovascular events than clinic BP in patient populations (Verdecchia et al., 1994). CKD patients have been shown to have higher SBP at night and display a non‐dipping pattern of nocturnal SBP more frequently compared to hypertensive individuals without CKD, independent of daytime or office BP control (Farmer et al., 1997; Peixoto & White, 2002). In our cohort of CKD patients with treated HTN, 75% of patients were identified as non‐dippers. This finding is in line with previous reports that demonstrated as high as 80% prevalence of non‐dipping pattern in patients with CKD, although a wide range of non‐dipping prevalence has been reported (25 ~ 80%) (Iimuro et al., 2013; Pogue et al., 2009), in part due to differences in comorbidity status and definitions used to define nocturnal dipping (Pogue et al., 2009; Wang et al., 2013).

Elevated nighttime BP is associated with more severe end‐organ damage including greater arterial stiffness and cardiac hypertrophy in hypertensive patients (Cuspidi et al., 2003, 2004). Similarly, elevated nighttime BP is a strong predictor of cardiovascular outcomes in CKD (Wang et al., 2016) as well as kidney disease progression (Fukuda et al., 2004; Paoletti et al., 2006). In CKD, non‐dipping has been shown to be an independent risk factor for target organ damage including coronary artery calcification and left ventricular hypertrophy (Choi et al., 2017; Jaques et al., 2018). In the current study, it is noteworthy that the office BP and daytime BP were similar between dippers and non‐dippers; the non‐dipping pattern in this cohort was exclusively explained by a higher nighttime BP (with a mean difference of + 16, 11, and 13 mmHg for SBP, DBP, and MAP, respectively). Interestingly, this nighttime‐dependent non‐dipping pattern in our participants contrasts with other studies in normotensive healthy individuals whose dipping magnitude was mainly due to lower daytime BP (Narkiewicz et al., 2002). The significant difference is only shown in nighttime BP, but not in the office and daytime BP, may highlight the importance of inclusion of ABPM for the detection and optimal management of HTN in clinical populations with high burden of nocturnal HTN, such as those with CKD.

Despite the high prevalence of non‐dipping in CKD, the mechanisms underlying elevated nighttime BP in CKD are not well understood. SNS overactivation, a characteristic of patients with reduced renal function, may be one contributing mechanism since the autonomic nervous system plays a major role in mediating the intrinsic circadian variation in BP (Somers et al., 1993). Prior studies have shown that diurnal variation in BP was abolished in patients with autonomic failure (Carvalho et al., 2000) and with cervical spinal cord injuries (Munakata et al., 1997). Increased SNS activation is not only linked to essential HTN (Grassi et al., 2018) but also implicated in abnormal diurnal BP regulation in otherwise normotensive individuals. In healthy individuals, norepinephrine and epinephrine levels show a diurnal variation with a decrease occurring during nighttime (Akerstedt, 1979; Linsell et al., 1985), while non‐dippers have reduced nighttime fall in both norepinephrine and epinephrine excretion rates as well as heighted α‐adrenergic receptor responsiveness compared to dippers (Sherwood et al., 2002). Similarly, hypertensive non‐dippers have higher plasma catecholamine levels (Kohara et al., 1995) and altered sympathovagal balance characterized by increased SNS and decreased parasympathetic activation compared to dippers (Hojo et al., 1997; Kohara et al., 1995). In hypertensive rats, administration of prazosin, an α‐adrenergic blocker, but not captopril or hydralazine, blunted the typical diurnal BP profile in hypertensive rats (Janssen et al., 1991; Oosting et al., 1997) highlighting the important role of vascular α‐adrenergic tone in mediating diurnal BP fluctuations (Panza et al., 1991). Similarly, in patients with HTN, nighttime dosing of α‐blockers reduced nighttime BP in non‐dippers (Kario et al., 2000), suggesting an important role of the SNS in modulating diurnal BP patterns.

Our results in CKD are aligned with previous data in other populations, suggesting that sympathetic neural mechanisms are likely involved in nighttime BP regulation and account for individual differences in nighttime BP dipping in CKD. We showed that MSNA, a direct measure of SNS outflow, is elevated in non‐dippers compared to dippers among nondiabetic CKD patients with treated HTN. In patients with HTN, Grassi et al. demonstrated an exaggerated MSNA in reverse dippers, a more severe form of the non‐dipping pattern defined as a day‐to‐night dipping ratio < 0% compared to dippers among patients (Grassi et al., 2008). While we classified our CKD cohort into dichotomous groups (dippers and non‐dippers) for the main analysis, we did perform a subgroup analysis in reverse dippers (*n* = 5) that showed that MSNA was, indeed, numerically higher in reverse dippers compared to non‐dippers without reverse dipping (*n* = 17) (84 ± 7 versus. 80 ± 14 bursts/100 HR, *p* = .545) and dippers (versus. 67 ± 13 bursts/100 HR, *p* = .036). These results were independent of OSA which is highly prevalent in patients with CKD and is also associated with SNS overactivation and nocturnal hypertension (Narkiewicz & Somers, 1997). There was no difference in the proportion of patients with OSA among dippers and non‐dippers, and analyses remained significant after adjusting for OSA, suggesting that SNS overactivation is linked to non‐dipping BP patterns in CKD, independent of OSA.

To further appreciate the relationship and the predictive value of MSNA on nighttime BP, we performed univariate correlation and multivariate regression analysis. These analyses demonstrated that MSNA was positively associated with nighttime as well as daytime SBP and DBP, and MSNA was an independent predictor for nighttime SBP and DBP, after adjustment for age, BMI, and smoking status. In hypertensive patients, Hering et al., demonstrated that MSNA was independently associated with HR after adjustment for age, BMI, and sex, but not with daytime and nighttime BP (Hering et al., 2011). The discrepancy between the prior findings in HTN and current findings in CKD may be due to the different disease states (HTN versus. CKD with HTN) and the lower daytime BP (~10 mmHg) and HR (~10 bpm) in the current study likely due to control of daytime BP with antihypertensive medications. In addition, we observed an inverse relationship between resting MSNA and the day‐to‐night BP difference in CKD. This is consistent with a previous report in hypertensive patients demonstrating that the greater the SNS activation, the less the magnitude of dipping at night (Grassi et al., 2008), although other studies have found no measurable difference in MSNA between dippers and non‐dippers in patients with HTN (Hering et al., 2011). In our cohort of CKD patients, we also showed using a step‐wise regression analysis that nighttime BP, as opposed to office BP or daytime BP, is more strongly linked to MSNA. These results are clinically relevant and may offer mechanistic insight into prior epidemiologic observations that have demonstrated that nighttime BP predicts future CVD and mortality better than traditional office BP and daytime BP measurements (Verdecchia et al., 1994).

CKD is also associated with markedly reduced NO bioavailability leading to endothelial dysfunction (Martens et al., 2016). Consistent with other groups (Kuczmarski et al., 2011), we previously reported reduced FMD, a measure of the endothelium‐dependent relaxation of vascular tone, in patients with CKD (Downey et al., 2017). Endothelial dysfunction in some studies has been linked to elevated nocturnal BP and nocturnal non‐dipping in hypertensive populations (Higashi et al., 2002) including uncontrolled HTN (Quinaglia et al., 2011). In contrast, Salles et al. showed that non‐dipping BP patterns were not associated with FMD, but were associated with endothelium‐independent vasodilation measured by nitroglycerin‐mediated vasodilation, in patients with resistant HTN (Fontes‐Guerra et al., 2015). The current study is the first to report in CKD patients that FMD is lower in non‐dippers and is inversely associated with nocturnal BP. In addition, similar to MSNA, FMD independently predicted nighttime BP in multivariate analyses, and there was a stronger association between FMD and nighttime BP levels (as opposed to daytime and office BP) in our CKD cohort. These results support the concept that nighttime BP regulation maybe mediated in part by vascular endothelial function in patients with CKD. Subgroup analyses to examine whether the contribution of sympathetic activity and vascular endothelial function in nighttime BP differs between dippers and non‐dippers can be investigated in further studies with bigger sample size.

In addition to its local vasodilatory effects, NO also has a central inhibitory effect on sympathetic outflow. Consistent with previous evidence demonstrating the link between NO‐metabolism and SNS (Charkoudian et al., 2006), we observed a strong association between FMD and MSNA in CKD. Our previous study demonstrated that 12 weeks of dietary supplementation of tetrahydrobiopterin, an essential co‐factor for NO synthesis, reduced MSNA in CKD patients (Park et al., 2015), demonstrating the potential interaction of NO‐dependent endothelial function and SNS in CKD. As described above, SNS overactivation and vascular endothelial dysfunction might each independently contribute to the abnormal nighttime BP regulation in patients with CKD. Moreover, MSNA and FMD may be interrelated in their influence on nighttime BP, considering their known physiologic interactions in neurocirculatory control. To address this, we performed a mediation analysis that showed that the mediating effect of MSNA on the association between FMD and nighttime BP is considerable; 47% of the relationship between FMD and nighttime BP was explained by the influence of MSNA burst incidence. Alternatively, 28% of the relationship between MSNA burse incidence and nighttime BP was explained by the influence of FMD although the statistical significance of this analysis indicated by the confidence intervals is marginal likely owing to the small sample size. Therapeutic interventions targeting endothelial dysfunction in CKD may have the potential to improve elevated nighttime BP by directly improving endothelial‐dependent vasodilatory capacity and/or by indirectly mitigating SNS overactivation which should be tested in future studies.

### 4.1 Limitations

We excluded CKD patients with comorbid diabetes since diabetes affects SNS regulation (Huggett et al., 2003). Therefore, results may not be generalizable to CKD patients with diabetes which is commonly comorbid with CKD. A small percentage in each group had comorbid OSA which is known to be associated with SNS overactivation and nocturnal hypertension (Narkiewicz & Somers, 1997). However, there was no difference in the proportion of OSA between groups, and results remained significant after adjusting for OSA. Similarly, although there was no significant difference in the proportion of smokers between the groups, smokers were identified only in the non‐dipping group. Smokers abstained from smoking for at least 12 hr before physiologic measures, and there was no effect of smoking status on daytime and nighttime BP, MSNA, and FMD within non‐dippers and in the entire cohort, consistent with prior studies that showed no difference in nocturnal dipping between smokers and non‐smokers (Morillo et al., 2006). Nevertheless, we included smoking status as a covariate for the regression‐based analysis. FMD was determined by measuring brachial artery diameter intermittently, rather than continuously, after cuff release; therefore, peak hyperemia may have occurred at a time point that was not captured in this technique. Although FMD measurements were performed uniformly in all subjects across time by a single investigator, continuous measurements of arterial diameter may have improved the detection of peak hyperemia and normalization of FMD by shear rate. Resting MSNA and FMD were performed in the supine position during daytime, not during nighttime. Nonetheless, daytime MSNA measures were strongly associated with nighttime BP (more strongly so than with daytime BP or office BP) in the current study, suggesting that daytime or global SNS activation is linked to nighttime hemodynamics and diurnal variation. Last, only men were included in this study and thus, the results cannot be extrapolated to both sexes. Future studies should include both sexes and perform sex‐stratified analyses to confirm our results.

## 5 CONCLUSION

HTN is an important risk factor for the progression of kidney disease, CVD, and mortality in CKD. To date, diagnosis and management of HTN have almost exclusively relied on office or daytime BP measurements. However, the current results suggest that non‐dipping and nocturnal HTN is highly prevalent in CKD and would otherwise have not been diagnosed without ABPM given the similarities in daytime and clinic BP readings between the non‐dipping and dipping groups. These data support our hypothesis that SNS activity and vascular endothelial function are involved in the regulation of nighttime BP as well as the magnitude of BP drop at nighttime in CKD patients. SNS overactivity and reduced NO bioavailability may be mechanistic pathways contributing to abnormal ABPM patterns, as well as mechanisms by which nocturnal hypertension leads to greater CVD risk. Further studies are warranted to determine the causal relationship of elevated nocturnal BP with SNS overactivity and endothelial dysfunction and whether therapeutic strategies targeting the SNS and NO metabolism could modify nocturnal HTN and long‐term cardiovascular risk in patients with CKD.

## CONFLICT OF INTEREST

No conflicts of interest, financial or otherwise, are declared by the author(s).

## AUTHOR CONTRIBUTIONS

J.J. and J.P. conceived and designed research; D.D., A.A.Q. and J.P. performed experiments; J.J., I.T.F., and J.P. analyzed the data; J.J., I.T.F., A.A.Q., and J.P. interpreted the results of experiments; J.J. and J.P. prepared figures; J.J. and J.P. drafted the manuscript; J.J., I.T.F., A.A.Q., D.D., and J.P. edited and revised the manuscript; J.J., I.T.F., A.A.Q., D.D., and J.P. approved the final version of the manuscript.
